# Usefulness of New Neutrophil-Related Hematologic Parameters in Patients with Myelodysplastic Syndrome

**DOI:** 10.3390/cancers15092488

**Published:** 2023-04-26

**Authors:** Iwona Kwiecień, Elżbieta Rutkowska, Krzysztof Gawroński, Katarzyna Kulik, Alicja Dudzik, Agata Zakrzewska, Agata Raniszewska, Waldemar Sawicki, Piotr Rzepecki

**Affiliations:** 1 Laboratory of Hematology and Flow Cytometry, Department of Internal Medicine and Hematology, Military Institute of Medicine-National Research Institute, Szaserów 128 Street, 04-141 Warsaw, Poland; 2 Department of Internal Medicine and Hematology, Military Institute of Medicine-National Research Institute, Szaserów 128 Street, 04-141 Warsaw, Poland

**Keywords:** myelodysplastic syndrome, hematology analyzer, neutrophil scattering items, Sysmex, cytogenetic changes, neutrophil parameters, monocyte-related parameter, immature granulocytes

## Abstract

**Simple Summary:**

The assessment of the new hematological neutrophil- and monocyte-related parameters may be important in myelodysplastic syndrome (MDS) patients for rapid diagnostics. The aim of the study was to assess the new hematological parameters in the bone marrow of MDS patients with and without cytogenetic changes. A total of 45 patients with MDS, including 17 patients with cytogenetic changes, were examined. The study was conducted using the Sysmex XN-Series hematological analyzer. New neutrophil and monocyte data relating to granularity, activity, volume and maturity (NE-WX/MO-WX, NE-WY/MO-WY, NE-WZ/MO-WZ, MO-X, MO-Y, MO-Z, and IG) were evaluated. We observed higher median proportions of NE-WX, NE-WY, NE-WZ, and IG count in MDS patients with cytogenetic changes than in patients without cytogenetic changes. The NE-FSC parameter was lower in MDS patients with cytogenetic changes than in patients without cytogenetic changes. New neutrophil-related parameters are shown to be useful in the rapid detection of patients with cytogenetic changes.

**Abstract:**

Myelodysplastic syndromes (MDS) are common malignant disorders with a poor prognosis. It is necessary to search for new rapid diagnostic methods to detect MDS patients with cytogenetic changes. The aim of the study was to assess new hematological neutrophil- and monocyte- related parameters I then bone marrow of MDS patient with and without cytogenetic changes. A total of 45 patients with MDS, including 17 patients with cytogenetic changes, were examined. The study was conducted using the Sysmex XN-Series hematological analyzer. New neutrophil and monocyte parameters, such as immature granulocytes (IG), neutrophil reactivity intensity (NEUT-RI), neutrophil granularity intensity (NEUT-GI), neutrophil size (NE-FSC) and neutrophil/monocyte data relating to granularity, activity and volume (NE-WX/MO-WX, NE-WY/MO-WY, NE-WZ/MO-WZ, MO-X, MO-Y, MO-Z) were evaluated. We observed higher median proportions of NE-WX, NE-WY, NE-WZ, and IG counts in MDS patients with cytogenetic changes than in patients without cytogenetic changes. The NE-FSC parameter was lower in MDS patients with cytogenetic changes than in patients without cytogenetic changes. The combination of new neutrophil parameters was found to be a new successful approach in distinguishing MDS patients with cytogenetic changes from patients without cytogenetic changes. It appears that there may be unique neutrophil parameter signatures associated with an underlying mutation.

## 1. Introduction

Myelodysplastic syndromes (MDS) are clonal disorders in which the bone marrow does not produce enough healthy blood cells [1]. MDS is usually associated with mutations or chromosomal aberration that affect the production and differentiation of blood cells leading to cytopenias [2]. Some common genetic abnormalities found in MDS mutations in genes such as TP53, ASXL1, and DNMT3A together with other mutations might result in clonal expansion affecting the function of cells and contributing to the development and progression of MDS [3,4,5]. Chromosomal abnormalities such as deletions or duplications are relatively common in MDS, having been detected in up to 60–70% of cases with the most common deletions being of chromosome 5, chromosome 7, and the long arm of chromosome 17 (known as 17q) [6]. Deletions or structural abnormalities of these chromosomes are associated with poorer prognosis, increased risk of clinical evolution and the development of acute myeloid leukemia (AML) [7].

Other chromosomal abnormalities that are seen in MDS include translocations, inversions, and duplications of genetic material. These changes can affect the structure and function of genes, and may also contribute to the development and progression of MDS [8]. There is a need for more rapid and non-invasive diagnostic methods that can accurately identify MDS and its specific subtype. One potential approach is the use of molecular tests that can detect specific genetic mutations or abnormalities associated with MDS. Some of the current research efforts in this area include the use of next-generation sequencing (NGS) technology to rapidly sequence and analyze genetic material from blood or bone marrow samples [9]. NGS can identify specific mutations and chromosomal abnormalities associated with MDS, and may also help predict disease progression and response to treatment [10]. However, this method is expensive and not widely available. The new hematology blood cell analyzer provides fast and accurate results for a wide range of blood and bone marrow parameters, including new neutrophil- and monocyte-related parameters [11]. These new parameters have been shown to be useable in the diagnosis and monitoring of various hematological disorders, including leukemias and lymphomas [12,13]. In particular, they have been reported to be useful in the early detection of sepsis and in the differentiation of bacterial and viral infections [14,15,16]. Regarding their use in MDS, there is limited research available so far.

In this article, we hypothesized that new research parameters associated with changes in the abundance and function of monocyte and neutrophils may be associated with the development and progression of MDS. The aim of the study was to assess the new hematological neutrophil- and monocyte-related parameters in the bone marrow of MDS patient with and without cytogenetic changes.

## 2. Materials and Methods

### 2.1. Study Participants

The study group consisted of 45 patients with suspicion of MDS, 17 with cytogenetic abnormalities and 28 without cytogenetic changes. Samples were collected from 10 May 2021 to 1 December 2022 at the Military Institute of Medicine-National Research Institute (Department of Internal Medicine and Hematology, Laboratory of Hematology and Flow Cytometry).

Peripheral blood (PB) samples and bone marrow (BM) aspirates samples were collected from all patients. Hematological parameters were measured on PB samples and BM samples collected in EDTA-K3 tubes (Beckton Dickinson, Franklin Lakes, NJ, USA), processed within 2 h from the sample collection on a Sysmex XN-series hematology system (Sysmex Co., Kobe, Japan). Leukocyte distribution and also new Sysmex parameters were analyzed in BM. The description of new hematological parameters is presented in Table 1.

MDS patients’ characteristics including age, gender as well as clinical and laboratory data—white blood cells (WBC), hemoglobin (Hb), red blood cells (RBC), mean corpuscular volume (MCV), platelets (PLT) and absolute neutrophil number—are presented in Table 2.

Cytogenetic abnormalities were investigated in BM aspirates by classical cytogenetics and/or fluorescent in situ hybridization (FISH). Classical cytogenetics on direct 24 to 48 h BM cultures and analysis of G-banded chromosomes were evaluated. Patient’s DNA in the form of chromosomes in metaphasis were subjected to hybridization to detect numeric and structural abnormalities. Two hundred interphase nuclei were counted.

MDS was confirmed after analyzing data from complete blood cell count, cell population data and information about dysplastic neutrophil by microscopic examination. Patients with other hematologic malignancies, other solid cancers, infection, inflammation, acute or chronic diseases and medication were excluded.

The PB and BM samples used in the study were taken during routine diagnostics and were approved by the Ethics Committee of the Military Institute of Medicine and all patients gave informed consent (Military Institute of Medicine Ethics Committee protocol code 16/WIM/2021 date: 21 April 2021).

### 2.2. New Hematological Parameters Connected with Neutrophils and Monocytes

The measurement of structural and activation parameters of neutrophils and monocytes on the Sysmex XN-1500 analyzer is based on fluorescent flow cytometry. Cells are permeabilized and nucleic acids were labeled with a fluorescent dye. The intensity of frontally scattered fluorescent light indicates the size of the cell (FSC parameter), the intensity of fluorescent light laterally scattered fluorescent light shows internal structure and granularity of the cell (SSC parameter). The intensity of fluorescent light in the area of neutrophils and monocytes indicates the amount of DNA and RNA present in the cell (SFL parameter), which increase with the metabolic activity of cells [17].

The measurement signals related to FSC, SSC and SFL are analyzed and plotted on a scattergram.

The immature granulocyte (IG) fraction includes promyelocytes, myelocytes and metamyelocytes, and is arranged above the neutrophil population in the differential fluorescence (WBC) scattergram of white blood cells [18].

The NE-WY and MO-WY parameters are calculated based on the spread around the mean fluorescence value on the cell scattergram. They represent the range of fluorescence distribution for cells excluding values below 20% of the peak height of the distribution curve [19]. The NE-WZ/MO-WZ and NE-WX/MO-WX parameters are calculated analogously to NE-WY/MO-WY and correspond the width of dispersion of neutrophil size and complexity, respectively.

The MO-X, MO-Y, and MO-Z parameters assess the intensity of laterally scattered light, dispersed in the monocyte region and frontal diffuse light, indicating cell complexity, activation, and monocyte size [20].

All new parameters collected are summarized in the Table 1.

### 2.3. Statistical Analysis

Statistica 13.0 software (TIBCO Software, Palo Alto, CA, USA) was used for a statistical analysis. For group comparison, Mann–Whitney U tests were used. A level of *p* < 0.05 was considered as statistically significant. The results are expressed as geometric mean (standard deviation) for demographic and laboratory data and medians (Q1–Q3) for the analyzed parameters.

## 3. Results

The diagnosis of MDS relied on a bone marrow aspirate for enumeration of blasts, the assessment of dysplasia, and the number of ringed sideroblasts, and cases were classified according to the WHO classification Revised 4th Edition [21]. The distribution of cases was as follows: 1 with 5q deletions (5q−), 1 with ring sideroblasts and single lineage dysplasia (MDS-RS-SLD), 1 MDS with excess blast type 1 (MDS-EB-1), 5 with ring sideroblasts and multilineage dysplasia (MDS-RS-MLD), 16 MDS with single lineage dysplasia (MDS-SLD), and 21 MDS with multilineage dysplasia (MDS-MLD). Patients were divided into two groups: with cytogenetic changes (*n* = 17) and without cytogenetic changes (*n* = 28). The clinical characteristics of the investigated groups with MDS are summarized in Table 2.

Bone marrow counts, such as WBC, neutrophils, lymphocytes, monocytes, eosinophils, basophils counts and IG, were analyzed for the two groups of MDS patients.

**Figure A1:**
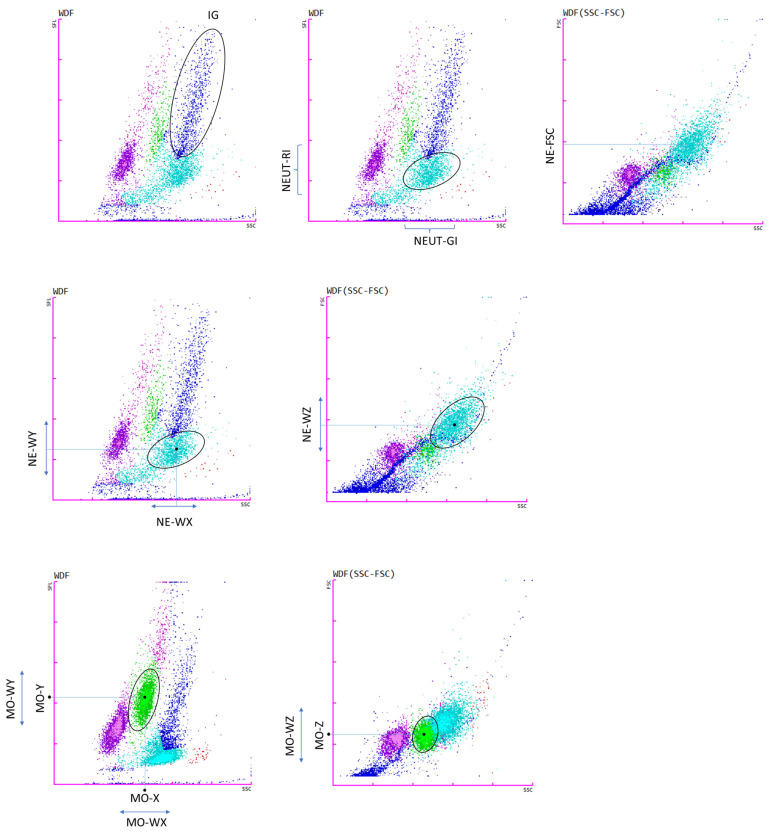
Sample screenshot scattergrams from the Sysmex XN-1500 analysis showing: immature granulocytes (IG); neutrophil reactivity intensity parameter (NEUT-RI) indicated by increased fluorescence intensity, relative shift from blue line; neutrophil granularity intensity (NEUT-GI), relative shift from blue line; NE-WX/MO-WX reflects the width of dispersion of cells population respectively for neutrophils or monocytes, with respect to cell side-scatter (SSC); NE-WY/MO-WY represents the fluorescence distribution width of cell populations, for neutrophils or monocytes, NE-WZ/MO-WZ reflects the distribution width of cell populations, proportional to the width of dispersion of cell size, for neutrophils or monocytes; NE-FSC reflects neutrophils cell size, neutrophil forward scatter mean intensity; MO-X depends on cell complexity, MO-Y Represents the mean value of fluorescence intensity; MO-Z indicates cell size, with respect to cell forward scatter (FSC). The scattergrams were plotted using side scatter (SSC, intracellular structure) on the *x*-axis and the side fluorescence signal (SFL, presence of nucleic acid) on the *y*-axis or forward scatter (FSC, mean intensity, reflects cell size) on the *y*-axis. Each dot represents one cell.

Additionally, new parameters connected with neutrophils and monocytes were evaluated using the Sysmex research parameters (parameter characteristics in Table 1 in the Section 2). A sample screenshot from the Sysmex XN-1500 analysis software showing selected neutrophil- and monocyte-related parameters is presented in Appendix A, Figure A1.

### 3.1. Cytogenetic Characteristics of Study Groups

**Figure 1:**
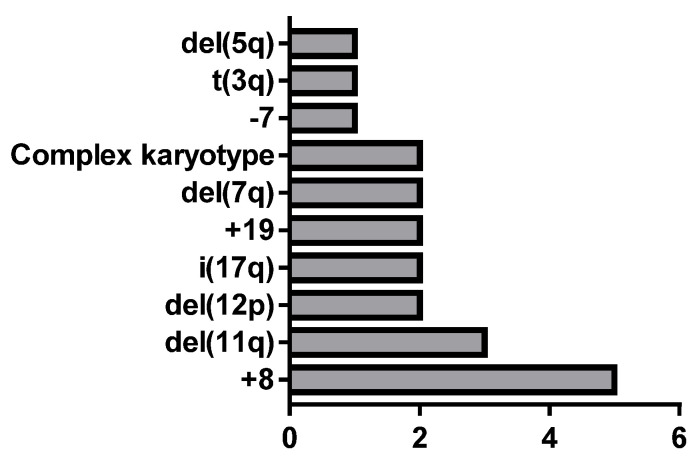
Chromosome abnormalities in MDS patients. Data expressed as number of patients with each abnormality.

Of the 45 MDS cases, all were subjected to chromosome analysis, and chromosome abnormalities were observed in 17 cases (Appendix A, Table A1). The frequency of abnormalities is shown in Figure 1. In the 17 MDS cases with chromosomal abnormalities, 5 cases of +8 and 3 cases of del(11q) were identified, and these occurred significantly more often than other chromosomal abnormalities (*p* < 0.05).

### 3.2. Leukocytes Characteristic of MDS Patients with and without Cytogenetic Changes

There was a higher median proportion of absolute neutrophil count in patients with cytogenetic changes than in patients without cytogenetic changes (19.3 vs. 8.0 × 10^3^/µL, respectively; *p* = 0.016302). In this study we observed significantly higher median proportion of absolute lymphocyte counts in patients with cytogenetic changes than in patients without cytogenetic changes (4.3 vs. 2.5 × 10^3^/µL, respectively; *p* = 0.006918). There was a higher median proportion of absolute monocyte counts and monocytes percentage in patients with cytogenetic changes than in patients without cytogenetic changes (3.3 vs. 0.9 × 10^3^/µL; *p* = 0.005343 and 10.5 vs. 7.1; *p* = 0.032669, respectively). We noted a higher median proportion of absolute basophil counts in patients with cytogenetic changes than in patients without cytogenetic changes (0.5 vs. 0.2 × 10^3^/µL, respectively; *p* = 0.007126). The median proportion of absolute immature granulocyte counts was also higher in patients with cytogenetic changes than in patients without cytogenetic changes (7.4 vs. 2.3 × 10^3^/µL, respectively; *p* = 0.016302) (Table 3).

### 3.3. New Parameters Connected with Neutrophils

**Figure 2:**
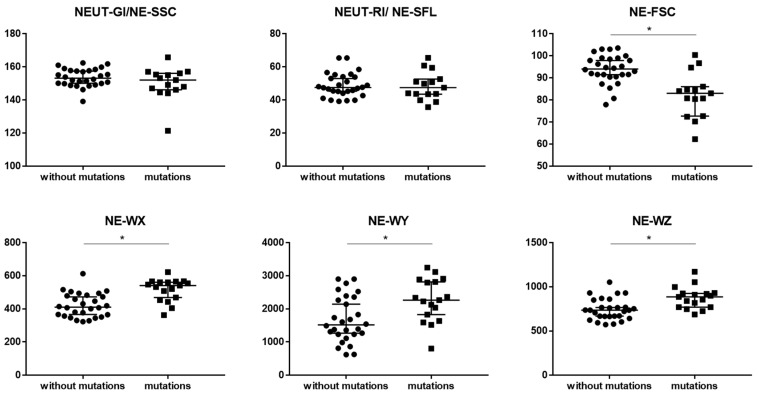
The differences between MDS patients with mutations and without mutations for neutrophil Sysmex parameters. Graphs show the median values (−). Significant differences for the analyzed parameters between MDS patients with mutations and without mutations in the Mann–Whitney U test presented as * (*p* < 0.05). Abbreviation: NEUT-RI (NE-SFL)—neutrophil reactivity intensity (neutrophil fluorescence intensity); NEUT-GI (NE-SSC) —neutrophil granularity intensity (neutrophil complexity); NE-FSC—neutrophil size; NE-WX—width of dispersion of neutrophil complexity; NE-WY—width of dispersion of neutrophil fluorescence; NE-WZ—width of dispersion of neutrophil size.

There was lower median proportion of NE-FCS parameter in patients with cytogenetic changes than in patients without cytogenetic changes (83.0 vs. 94.1, respectively; *p* = 0.000278). We observed higher median proportions of NE-WX, NE-WY and NE-WZ parameters in patients with cytogenetic changes than in patients without cytogenetic changes (541.0 vs. 410.5; *p* = 0.000099, 2266.0 vs. 1517.0; *p* = 0.008657, and 887.0 vs. 736.0; *p* = 0.000988, respectively) (Table 4 and Figure 2). No statistically significant differences were observed for the NEUT-RI and NEUT-GI parameters between patients with and without cytogenetic changes.

### 3.4. New Parameters Connected with Monocytes

**Figure 3:**
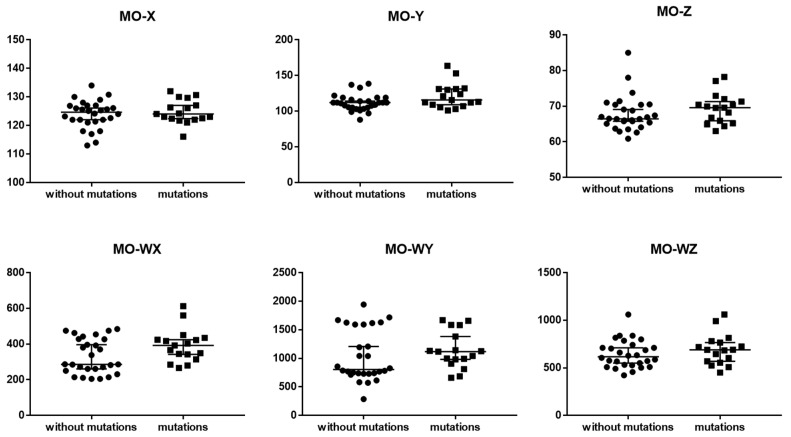
The differences between MDS patients with mutations and without mutations for monocytes Sysmex parameters. Graphs show the median values (−). Abbreviation: MO-X—monocyte complexity; MO-Y—monocyte fluorescence; MO-Z—monocyte size; MO-WX—width of dispersion of monocytes complexity; MO-WY—width of dispersion of monocyte fluorescence; MO-WZ—width of dispersion of monocyte size.

Taking into account the new hematological parameters connected with monocytes such as MO-X, MO-Y, MO-Z, MO-WX, MO-WY and MO-WZ, no statistically significant differences were observed between the groups of MDS patients with and without cytogenetic changes (Table 4 and Figure 3).

## 4. Discussion

In this study, we focused on the assessment of new neutrophil and monocyte parameters to characterize and distinguish MDS patients with cytogenetic changes and without cytogenetic changes. Firstly, we analyzed the PB morphology of MDS patients and cytogenetic abnormalities to characterize study groups. Chromosome aberrations were observed in 17 cases, +8 and del (11q) were the most common, and significantly more frequent than other chromosomal abnormalities such as: del(5q), t(3q), −7, complex karyotype, del(7q), +19, i(17q), del(12p), and del(12p). In the literature, the most frequent cytogenetical abnormalities detected by conventional karyotyping among MDS are also −7/del(7q) and −5/del(5q), followed by +8, dup(1q), del(20q), del(11q), del(12p)/t(12p), del(17p)/iso(17q), as well as del(18q), +21q gains, del(13q), and +der(1;7)(q10;p10) [22].

Next, we characterized the BM main leukocyte subpopulation in MDS patients using a hematological analyzer. We observed higher median proportions of absolute neutrophil, lymphocyte, monocyte and basophil counts in patients with cytogenetic changes than in patients without cytogenetic changes. In high-risk MDS patients, peripheral cytopenia is visible despite BM hypercellularity. The dysplastic cells produced in BM do not function properly and are destroyed by apoptosis [23].

### 4.1. New Parameters Connected with Neutrophils

In our study, the median proportion of the IG count was higher in patients with cytogenetic changes than in patients without cytogenetic changes. The available results suggest that the IG count may be important prognostic marker in patients with MDS. Specifically, a higher IG count has been associated with specific cytogenetic changes. We found no studies evaluating IG levels in the BM of MDS patients.

Lu et al. [24] showed that the IG assessment is important in the diagnosis of myeloid neoplasms (MN), comparing the IG estimation on a hematology analyzer with manual score. Blood samples from 388 patients were analyzed. There was a high level of agreement derived from XN and manual measurements in MN patients, but only a moderate correlation for patients with acute myeloid leukemia (AML). IG parameters from the Sysmex XN analyzer are helpful in MN screening, although granulocyte morphological abnormalities may interfere with the accuracy of IG parameters.

Mishra et al. [25] presented a case report about the prognostic value of IG % in a patient with chronic myeloid leukemia (CML). The study found that IG fraction elevated at diagnosis decreased after treatment, which was confirmed by microscopic examination. This article highlights the importance of the IG fraction as a guide for the first suspicion of early CML in a young patient and its possible role in monitoring response to treatment.

Another study determined the usefulness of IG measurement as a predictor of infection or positive blood culture. Automated GI measurements have been shown to reflect biologically and clinically relevant phenomena, but are not sensitive enough to be used as screening tests to predict infection or bacteremia. However, a very high percentage was a specific predictor of sepsis and may help to expedite the laboratory microbiological evaluation of a subgroup of patients [26].

Based on the above information, we confirmed that IG assessment may serve as a robust prognostic marker in patients with MDS, especially in those with chromosomal changes. However, further studies are required to explore the potential clinical utility of using the IG parameter to assess disease severity and predict outcomes in MDS patients with chromosomal changes. While IG count may be a practical parameter for evaluating MDS patients with cytogenetic changes, it should be used in conjunction with other clinical and laboratory parameters to make a diagnosis and assess disease severity.

The evaluation of many neutrophil-related parameters during the verification of patients with MDS can provide important diagnostic and prognostic information. In addition to the IG, other parameters related to neutrophils that may be evaluated (and we analyzed in this study) include: NEUT-GI (NE-SSC), NEUT-RI (NE-SFL), NE-FSC, NE-WX, NE-WY, and NE-WZ. In our study we observed higher median proportions of the NE-WX, NE-WY and NE-WZ parameters in patients with cytogenetic changes than in patients without cytogenetic changes, and there was lower median proportion of the NE-FCS parameter in patients with cytogenetic changes than in patients without cytogenetic changes. We did not observe differences for the tested parameters NEUT-GI (NE-SSC) and NEUT-RI (NE-SFL) in MDS patients with and without cytogenetic changes.

There are several studies that have investigated the diagnostic and prognostic utility of the NE-WX, NE-WY, and NE-WZ parameters in various hematological disorders and infections. In a previous study, we observed the highest proportion of NE-WX, NE-WY, and NE-WZ parameters in healthy controls, without differences between a group of patients with SARS-CoV-2 infection and a convalescent group [14].

The usefulness of some structural parameters of white blood cells which are measured by the Sysmex analyzer have not widely been tested in MDS patients. There are several reports on this subject. Furundarena et al. determined the values of NEUT-X and NEUT-Y in the normal population and patients with MDS. The NE-X and NE-Y values in MDS patients with hypogranulated bone marrow were significantly lower than for MDS patients without hypogranulation. The authors suggested that the NE-X and NE-Y parameters can be used to detect neutrophil dysplasia in MDS and chronic myelomonocytic leukemia (CMML) patients [27]. Another article reported on structural blood cell parameter NEUT-X determined by the Sysmex analyzer in detecting MDS. The NE-X parameter with the granularity index could be used as an indicator of MDS before anemia [13].

Other researchers have pointed to the usefulness of MDS screening based on data from an automated hematology analyzer. Neutrophil volume and complexity parameters originating from peripheral blood may be suitable for detecting MDS patients [28]. Other data also indicate that new generations of hematology analyzers provide cell population data that can be used to reliably detect MDS from routine leukocyte composition determination [29]. Schillinger et al. found that the combination of parameters coming from new hematology analyzers can help exclude the diagnosis of CMML and thus reduce the number of blood smears tested in reactive monocytosis [12].

The abovementioned parameters from new hematology analyzers providing additional information about neutrophil morphology and heterogeneity may raise suspicion of MDS [20]. As demonstrated by the studies, these parameters have shown promise in their diagnostic and prognostic value in various hematological disorders.

Cell morphology should be quite variable according to the disease subtypes in MDS. For instance, the sizes and morphologies of blood cells are very unique in 5q- syndrome [30]. The morphologic patterns of blood cells should be widely different between MDS-SLD (single lineage dysplasia) and MDS-MLD (multilineage dysplasia) [31]. In the future, after increasing the number of patients, we plan to examine the utility of this new system for neutrophil-related parameters in each MDS subtype according to the WHO classification.

We compared new hematological parameters related to neutrophils and monocytes for the two most numerous groups, i.e.,: MDS-SLD (*n* = 16) and MDS-MLD (*n* = 21). No statistically significant differences were found (not shown in the text). The limitation of this study is the number of patients. We were unable to perform a reliable statistical analysis considering the new hematological parameters under the MDS subtypes.

After expanding the group, we plan to divide patients into subgroups and compare the relationship between new hematological parameters and changes in bone marrow cytology. However, at this stage, the assumption of the work was to determine whether the new diagnostic parameters related to monocytes and neutrophils can be at all important in distinguishing patients with cytogenetic changes from patients without cytogenetic changes in the entire study group, as a rapid screening test.

To summarize, a comprehensive evaluation of multiple parameters related to neutrophils, including the above parameters and neutrophil count, may provide a more complete picture of disease severity and prognosis in patients with MDS.

### 4.2. New Parameters Connected with Monocytes

Evaluating new monocyte-related parameters in MDS patients could potentially provide valuable insights into disease pathogenesis and help to identify patients who may benefit from more aggressive treatments. Monocytes are an important component of the immune system, and abnormalities in their numbers and function have been implicated in the development and progression of several hematological malignancies, including MDS [32]. However, in this study, no statistically significant differences were observed between the groups of MDS patients with and without cytogenetic changes for the MO-WX, MO-WY, MO-WZ, MO-X, MO-Y, and MO-Z parameters.

There is limited research available on the diagnostic and prognostic utility of the above parameters in hematological disorders, including MDS.

Di Luise et al. [20] investigated the diagnostic value of the monocyte-related parameters in patients with macrocytic anemia and myelodysplastic syndrome. It was observed that MO-X and MO-Y had statistically higher values in the group of MDS patients when compared with healthy controls and fewer high values when compared with the group of vitamin deficiency anemia patients. However, the authors highlight that significant differences in MO-X and MO-Y parameters may be a consequence of abnormalities in the nuclear and cytoplasmic morphology of monocyte granulations and the possible presence of promonocytes in the circulation, which is typical of MDS.

Given the limited research available on the MO-WX, MO-WY, MO-WZ, MO-X, MO-Y, and MO-Z parameters in hematological disorders, including MDS, it is difficult to draw any definitive conclusions about their diagnostic and prognostic value. However, the available studies suggest that these parameters may have potential as prognostic markers in other hematological conditions such as AML [33], and as diagnostic markers for bacterial infections [34]. Further research is needed to confirm their diagnostic and prognostic value and determine how they can be integrated into clinical practice.

The utility of this new system for neutrophil-related parameters should also be evaluated according to division into groups with different cytogenetic changes and to the grade of IPSS staging in order to determine its clinical utility. This would certainly be useful in the future for the precise characterization of patients with cytogenetic changes and for assigning patients to a specific MDS risk group. The current study group is too small to allow such divisions. At this stage, we have shown new parameters in the entire group of MDS patients in order to quickly identify patients in the first step of the diagnostic process.

## 5. Conclusions

In conclusion, the results of this work highlight the usefulness of the application of parameters provided by Sysmex analyzers in the detection of patients with MDS. We suggest that the NE-WX, NE-WY, NE-WZ, and NE-FSC markers could be included in routine BM research to differentiate patients with MDS and stratify risk groups. However, it is important to note that these research parameters are still being studied and validated, and their clinical utility in routine practice has not yet been established.

## Figures and Tables

**Table 1:** Description and significance of new neutrophil and monocytes parameters.

Parameters	Parameters Description
IG (Immature Granulocytes)	The IG fraction includes promyelocytes, myelocytes and metamyelocytes (without blasts and band cells)
NE-SFL (NEUT-RI) (Neutrophil Reactivity Intensity)	Represents the mean value of fluorescence intensity and increases in proportion to the content of nucleic acids in the cell. This reflects metabolic activity.
NE-SSC (NEUT-GI) (Neutrophil Granularity Intensity)	Dependent on neutrophil complexity. Increases in the presence of cytoplasmic granulation or vacuoles.
NE-WX/MO-WX	Reflects the width of dispersion of cells population, with respect to cell side-scatter (SSC).
NE-WY/MO-WY	Represents the fluorescence distribution width of the cell population, with respect to cell fluorescence intensity (SFL).
NE-WZ/MO-WZ	Reflects the distribution width of the cell population, with respect to cell forward scatter (FSC). It is proportional to the width of dispersion of cell size.
NE-FSC	Neutrophil forward scatter mean intensity. Reflects neutrophil cell size.
MO-X MO-Y MO-Z	Depends on cell complexity, presence of granularity and vacuoles. Represents the mean value of fluorescence intensity. Indicates cell size, with respect to cell forward scatter (FSC).

**Table 2:** Demographic and laboratory data of MDS patients.

	All Study Grup	MDS Patients without Mutations	MDS Patients with Mutations
n	45	28	17
Sex: f/m (n)	23/22	16/12	7/10
Age (mean ± SD years)	66 ± 14		
Women (mean ± SD years)	61 ± 14	67 ± 13	70 ± 13
Men (mean ± SD years)	59 ± 13	61 ± 16	71 ± 14
Blood hematological parameters (mean ± SD years)			
WBC [×10^3^/µL]	5.8 ± 4.3	6.1 ± 5.3	5.3 ± 1.9
RBC [×10^6^/µL]	3.1 ± 4.3	3.2 ± 0.7	3.0 ± 0.7
Hb [g/dL]	10.0 ± 1.9	10.1 ± 1.9	9.8 ± 2.0
MCV [fL]	96.3 ± 8.8	95.6 ± 9.3	97.3 ± 8.1
PLT [×10^3^/µL]	185 ± 124	184 ± 124	187 ± 128
Neutrophils [×10^3^/µL]	3.3 ± 3.2	3.6 ± 3.9	2.8 ± 1.7

Abbreviations: MDS: myelodysplastic syndrome; f: female; m:man; WBC, white blood cell count; RBC: red blood cells; Hb: haemoglobin; MCV: mean corpuscular volume; PLT, platelets.

**Table 3:** Median proportion of bone marrow cells between MDS patients: without mutations and with mutations. Data expressed as median (Q1-Q3). (* *p* < 0.05 Mann–Whitney U test).

	MDS Patients without Mutations Median (Q1–Q3)	MDS Patients with Mutations Median (Q1–Q3)	* *p* < 0.05 Mann–Whitney (U Test)
Neutrophils [10^3^/µL]	8.0 (5.1–13.0)	19.3 (8.9–27.5)	* 0.016302
Lymphocytes [10^3^/µL]	2.5 (1.9–3.5)	4.3 (3.1–5.3)	* 0.006918
Monocytes [10^3^/µL]	0.9 (0.4–1.6)	3.3 (0.9–4.9)	* 0.005343
Eosinophils [10^3^/µL]	0.3 (0.1–0.6)	0.7 (0.2–1.1)	0.066356
Basophils [10^3^/µL]	0.2 (0.1–0.3)	0.5 (0.2–0.9)	* 0.007126
Immature Granulocytes [10^3^/µL]	2.3 (0.6–4.5)	7.4 (2.9–9.7)	* 0.016302
Neutrophils [%]	65.3 (62.8–71.3)	66.5 (62.2–73.9)	0.561047
Lymphocytes [%]	20.9 (16.5–25.8)	18.1 (11.3–20.7)	0.158842
Monocytes [%]	7.1 (5.4–10.2)	10.5 (7.0–14.2)	* 0.032669
Eosinophils [%]	1.8 (0.9–4.0)	2.0 (1.1–2.4)	0.920524
Basophils [%]	1.4 (1.0–1.9)	1.7 (1.5–2.4)	0.245649
Immature Granulocytes [%]	19.1 (8.8–21.9)	23.1 (18.5–25.5)	0.101854
Blasts [%]	1.2 (1.0–2.3)	1.2 (1.0–1.7)	0.793117

Abbreviation: MDS—myelodysplastic syndrome.

**Table 4:** Median proportion of new neutrophil and monocyte parameters in MDS patients without mutations and with mutations. Data expressed as median (Q1-Q3). (* *p* < 0.05 Mann–Whitney U test).

	MDS Patients without Mutations Median (Q1–Q3)	MDS Patients with Mutations Median (Q1–Q3)	* *p* < 0.05 Mann–Whitney (U Test)
NEUT-GI (NE-SSC)	153.2 (149.9–157.7)	152.1 (146.2–156.2)	0.174810
NEUT-RI (NE-SFL)	47.5 (44.6–54.0)	47.5 (43.5–52.7)	0.696185
NE-FSC	94.1 (91.0–98.7)	83.0 (72.7–86.1)	* 0.000278
NE-WX	410.5 (361.0–481.0)	541.0 (469.0–561.0)	* 0.000099
NE-WY	1517.0 (1230.0–2312.5)	2266.0 (1828.0–2815.0)	* 0.008657
NE-WZ	736.0 (665.0–809.0)	887.0 (769.0–925.0)	* 0.000988
MO-X	124.6 (121.7–126.9)	124.0 (122.4–127.0)	0.685158
MO-Y	112.0 (105.0–117.6)	115.8 (109.0–131.2)	0.085358
MO-Z	66.4 (65.2–70.4)	69.6 (65.9–71.3)	0.204324
MO-WX	286.5 (255.5–427.5)	392.0 (342.0–424.0)	0.089794
MO-WY	805.5 (734.5–1593)	1118.0 (983.0–1383.0)	0.269285
MO-WZ	617.0 (529.5–726.0)	689.0 (569.0–765.0)	0.371312

Abbreviation: MDS—myelodysplastic syndrome; NEUT-RI (NE-SFL)—neutrophil reactivity intensity (neutrophil fluorescence intensity); NEUT-GI (NE-SSC) —neutrophil granularity intensity (neutrophil complexity); NE-FSC—neutrophil size; NE-WX—width of dispersion of neutrophil complexity; NE-WY—width of dispersion of neutrophil fluorescence; NE-WZ—width of dispersion of neutrophil size; MO-X—monocyte complexity; MO-Y—monocyte fluorescence; MO-Z—monocyte size; MO-WX—width of dispersion of monocytes complexity; MO-WY—width of dispersion of monocyte fluorescence; MO-WZ—width of dispersion of monocyte size.

## Data Availability

The data presented in this study are available in this article.

## Appendix A

**Table A1:** Characteristics of patients with chromosome abnormalities.

ID	Age	f/m	Chromosome Abnormalities	WBC [×10^3^/µL]	RBC [×10^6^/µL]	Hb [g/dL]	MCV [fL]	PLT [×10^3^/µL]	Neutrophils [×10^3^/µL]
1	63	m	del(11q)	4.84	3.26	12.3	110	62	2.09
2	72	m	del(7q) and i(17q)	2.75	2.37	7.3	93	45	0.44
3	70	m	−7	3.82	4.18	14.0	97	129	1.86
4	72	m	+19 and i(17q)	8.24	3.23	11.2	102	194	5.69
5	82	m	complex karyotype	5.75	2.99	10.5	104	180	2.08
6	44	m	del(11q)	2.87	4.66	12.9	83	79	1.5
7	52	f	t(3q)	5.50	3.57	8.9	82	446	3.2
8	61	m	complex karyotype	6.87	3.07	10.7	102	232	4.9
9	81	f	del(7q) and +8	3.48	2.31	7.1	90	53	1.67
10	75	f	del(11q)	8.06	3.39	10.4	89	245	5.12
11	64	f	+8	1.95	2.86	10.1	106	291	0.46
12	57	f	+8	5.80	2.45	9.0	107	411	2.9
13	64	m	del(3q)	7.87	2.99	9.5	102	273	5.42
14	88	m	del(12p)	6.00	2.23	7.1	95	186	2.16
15	75	f	+19 and +8	6.80	2.68	8.6	99	13	4.35
16	89	f	+8	4.43	2.74	8.1	93	60	1.72
17	92	m	del(5q) and del(12p)	4.32	2.54	8.6	100	280	2.14

Abbreviations: f: female; m:man; WBC, white blood cell count; RBC: red blood cells; Hb: haemoglobin; MCV: mean corpuscular volume; PLT, platelets.
